# Single-Nucleotide Polymorphisms Related to Multiple Myeloma Risk: A Systematic Review and Meta-Analysis

**DOI:** 10.3390/ijms26073369

**Published:** 2025-04-04

**Authors:** Giovanna Gilioli da Costa Nunes, Francisco Cezar Aquino de Moraes, Aline Beatriz Carvalho de Almeida, Felipe Goes Costa, Luiz Fernando Duarte de Andrade Junior, Maria Vitória Sabino Hupp, Ruan Rotondano Assunção, Marianne Rodrigues Fernandes, Sidney Emanuel Batista dos Santos, Ney Pereira Carneiro dos Santos

**Affiliations:** 1 Research Center of Oncology, Federal University of Pará Belém, Belém 66073-000, PA, Brazil; giovanna.nunes@ics.ufpa.br (G.G.d.C.N.); francisco.aquino.moraes@ics.ufpa.br (F.C.A.d.M.); aline.carvalho@icm.ufpa.br (A.B.C.d.A.); felipe.goes.costa@icm.ufpa.br (F.G.C.); luiz.junior@icm.ufpa.br (L.F.D.d.A.J.); maria.hupp@icm.ufpa.br (M.V.S.H.); fernandesmr@yahoo.com.br (M.R.F.); 2 Faculty of Medicine, Federal University of Pará Belém, Belém 66073-000, PA, Brazil; ruan.assuncao@ics.ufpa.br; 3 Laboratory of Human and Medical Genetics, Institute of Biological Science, Federal University of Pará Belém, Belém 66077-830, PA, Brazil; sidneysantosufpa@gmail.com

**Keywords:** multiple myeloma, epidemiology, risk, single-nucleotide polymorphism

## Abstract

Multiple myeloma ranks as the second most common hematopoietic malignancy in terms of both incidence and mortality. Prognostic stratification is critical for optimizing therapeutic strategies, as certain genetic alterations can significantly influence disease progression and treatment response. The meta-analysis analyzed data from 3421 multiple myeloma patients and 14,720 controls. PubMed, Web of Science, and Scopus were used as databases. Associations between the SNPs and multiple myeloma were calculated as a measure of pooled odds ratios (ORs) and 95% confidence intervals. Statistical analysis was performed using Review Manager (RevMan). *DNAH11* rs4487645 A/C genotype (OR = 1.35; 95% CI: 1.24–1.46; *p* < 0.00001; I^2^ = 0%), *ULK4* rs1052501 G/G genotype (OR = 1.21; 95% CI: 0.98–1.50; *p* = 0.08; I^2^ = 64%)*, ULK4* rs1052501 A/G genotype (OR = 1.23; 95% CI: 1.13–1.34; *p* < 0.00001; I^2^ = 0%), *DTNB* rs6746082 A/A genotype (OR = 1.10; 95% CI: 1.01–1.20; *p* = 0.03; I^2^ = 45%), and *VDR* rs1544410 A/G genotype (OR = 1.87; 95% CI: 1.04–3.36; *p* = 0.04; I^2^ = 0%) increased multiple myeloma risk. Our study concludes that *DNAH11*, *ULK4*, *DTNB*, and *VDR* may serve as predictive biomarkers for MM risk.

## 1. Introduction

Multiple myeloma (MM) is a hematological neoplasm characterized by the clonal proliferation of malignant plasma cells within the bone marrow, leading to the over-production of monoclonal immunoglobulins that contribute to end-organ damage, particularly affecting the bones and kidneys [1]. B cells, as essential components of humoral immunity, are responsible for antibody production during infectious processes, with plasma cells representing their terminal differentiation stage.

MM is a malignancy associated with high incidence and mortality rates, accounting for over 180,000 cases and 120,000 deaths worldwide annually, with the highest burdens reported in Asia, Europe, and North America [2]. The disease predominantly affects individuals over the age of 65 in industrialized nations, with notable risk factors including male sex, occupational exposure (e.g., firefighting), obesity, and exposure to dioxins or Agent Orange [3]. MM ranks as the second most common hematopoietic malignancy in terms of both incidence and mortality, constituting approximately 10% of all hematological cancers [4].

The clinical presentation of MM often encompasses anemia, leukopenia, thrombocytopenia, lytic bone lesions, hypercalcemia, and elevated serum creatinine. These features result from the pathological suppression of normal hematopoiesis, increased osteolysis, and renal impairment caused by the expansion of malignant plasma cells [3]. Diagnostic criteria include the presence of CRAB features—hypercalcemia, renal insufficiency, anemia, or bone lesions—or at least one myeloma-defining event (MDE), which may include clonal plasma cell infiltration ≥60%, a serum free light chain (FLC) ratio ≥100, or more than one focal lesion ≥5 mm detected on magnetic resonance imaging [5].

Research on the genetic underpinnings of MM has highlighted significant contributions of chromosomal abnormalities, particularly translocations involving chromosome 14, as well as the activation of oncogenes such as NRAS, KRAS, and BRAF [6]. However, despite these advancements, the etiology of MM remains incompletely understood. Prognostic stratification is critical for optimizing therapeutic strategies, as certain genetic alterations can significantly influence disease progression and treatment response [7].

The need for further investigation into the genetic determinants of MM is evident. Studies aimed at elucidating the genetic architecture of this malignancy could enhance prognostic accuracy, inform treatment protocols, and guide preventative measures.

This study aims to synthesize data from global literature to investigate genetic variants and candidate genes associated with increased susceptibility to MM. Specifically, this study focuses on evaluating the potential roles of DNAH11, VDR, DTNB, and ULK4 in the pathogenesis of MM, seeking to identify associations between these genetic elements and the epidemiological patterns observed globally.

## 2. Materials and Methods

### 2.1. Protocol and Registration

This systematic review followed the Preferred Reporting Items for Systematic Reviews and Meta-Analysis (PRISMA) guidelines [8]. The protocol was registered in the International Prospective Register of Systematic Reviews (PROSPERO) with registration number CRD42024620125.

### 2.2. Search Strategy

A comprehensive literature search was conducted to identify relevant studies published until 1 November 2024, in the PubMed, Scopus and Web of Science databases. The search strategy with the MeSH terms is detailed in Table S1, Supplementary Materials. Aiming at the inclusion of additional studies, the references of the included articles and systematic reviews of the literature were evaluated, and an alert was established for notifications in each database, in case a study corresponding to the consultation carried out was eventually published. Those found in the databases and in the references of the articles were incorporated into the reference management software (EndNote^®^, version X7, Thomson Reuters, Philadelphia, PA, USA). Duplicate articles were automatically and manually excluded. Titles and abstracts of articles found in the databases were analyzed independently by four reviewers (A.B.C.d.A., L.F.D.d.A., F.G.C. and M.V.S.H.). Disagreements were resolved by consensus between the two authors and the senior author (G.G.d.C.N., F.C.A.d.M. and N.P.C.S.).

### 2.3. Study Selection

The population, exposure, comparator, outcomes, and study design (PECOS) model was used to select potential studies: P (population), patients with MM; E (exposure) and C (comparator), SNPs related to MM risk and different genotypes; O (outcomes), susceptibility or greater risk of developing MM; and S (study design), observational (cohort, case-control, or cross-sectional).

Conference abstracts, preprints, theses, dissertations, and studies using biobanks were excluded. The inclusion criteria prioritized studies with diverse ancestral populations to identify genetic variants relevant across different groups. To ensure robust comparisons, only studies explicitly reporting the sample sizes of both patients and controls were included. Additionally, only significant variants were considered, and genotypes had to be present in all analyzed studies for these variants. Exclusion criteria involved studies that lacked information on sample sizes, reported non-significant results, or investigated SNPs unrelated to MM risk. This stringent selection process ensured the reliability and relevance of the findings, considering that the majority of available studies involve populations of European Caucasian ancestry.

Two reviewers (G.G.d.C.N. and F.C.A.d.M.) independently screened the titles and abstracts of citations to identify potentially relevant studies. Full-text articles were obtained, and the same two reviewers independently reviewed the articles according to the inclusion criteria. The third reviewer (R.R.A.) resolved any disagreements or doubts. This process was performed using Rayyan QCRI, a free web application designed to assist researchers in conducting systematic reviews.

### 2.4. Data Extraction

The following data were extracted by four independent reviewers (A.B.C.d.A., L.F.D.d.A., F.G.C. and M.V.S.H.) using standardized sheets in Microsoft Excel: authors; publication year; country where the study was conducted; total number; gender; ethnicity; mean age and mean age at diagnosis of participants with MM; genotyping method; histologically confirmed MM; and author’s main findings. Disagreements were resolved through discussion with the senior author (N.P.C.S.).

### 2.5. Quality Assessment

The Newcastle–Ottawa Scale (NOS) [9] was used to evaluate the methodological quality of the studies (risk of bias) by two groups of independent reviewers (G.G.d.C.N. and F.C.A.d.M.), and disagreements were resolved by discussion with another reviewer (R.R.A.). Three primary domains were evaluated in each study, namely, selection, comparability, and exposure. The maximum NOS scores for each domain were 4, 2, and 3 stars, respectively. Therefore, every study could attain a total score of 9. The Strengthening the Reporting of Genetic Association (STREGA) guidelines [10] were also used to assess the quality of reporting in the included studies. These guidelines comprise five main categories (genotyping methods and errors, population stratification, haplotype variation, Hardy–Weinberg equilibrium, and replication). The first category includes five items (genotyping platform, error and call rates, genotyping in batches, centers/laboratories of genotyping, and the number of individuals for successful genotyping). Thus, nine items were evaluated. To compare study quality, a total score was calculated by assigning one point to each item, with a higher score indicating better genetic study quality (range: 0–9). This instrument was applied by three independent reviewers (G.G.d.C.N., F.C.A.d.M. and R.R.A.), and disagreements were resolved by the senior author (N.P.C.S.).

### 2.6. Data Analysis

Detailed information about the SNPs was obtained from the genetic databases 1000 Genomes Project (1000 Genomes Project Consortium, 2015) [11] and *ClinVar* (Landrum et al., 2018) [12]. Statistical analyses were conducted using Review Manager (Rev Man), version 5.4.1 (The Cochrane Collaboration, Oxford, England). Associations between the SNPs and MM risk were calculated as a measure of pooled odds ratios (ORs) and 95% confidence intervals (CIs). We consider OR > 1 favoring increased risk of GBM and OR < 1 favoring decreased risk of GBM. Pooled OR was analyzed by the Mantel–Haenszel method (fixed-effect). The I^2^ range of 60–75% I^2^ was considered significant, indicating substantial heterogeneity. An I^2^ > 75% was considered to represent considerable heterogeneity.

## 3. Results

### 3.1. Search Results

**Figure 1:**
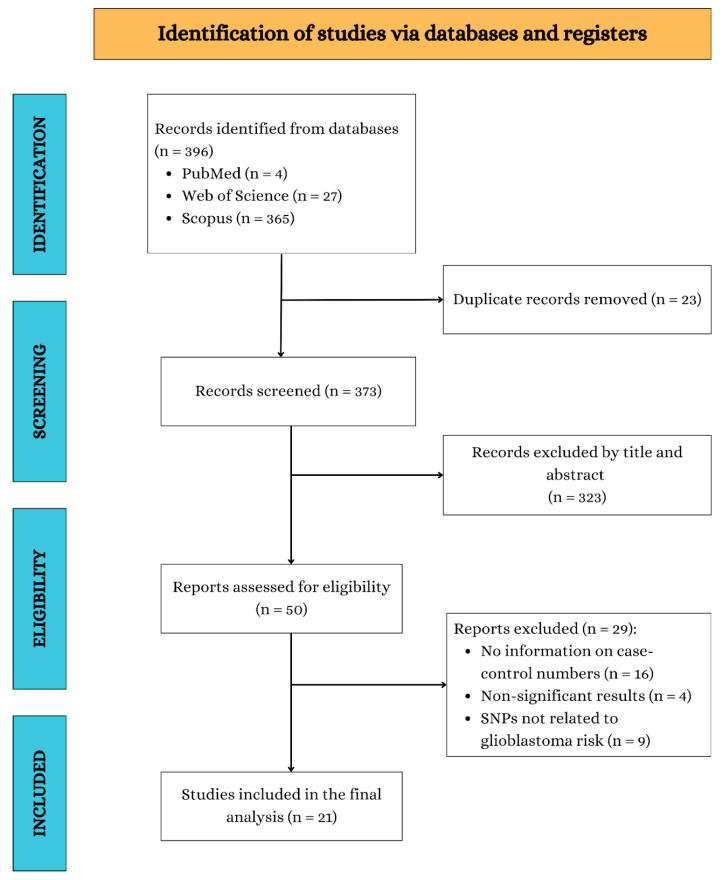
Study selection flowchart through literature search.

The electronic search identified 396 potentially relevant records. After removing duplicates, 373 records remain. Finally, after reviewing titles and abstracts, 21 articles were selected for full-text examination. Of these, only four met the inclusion criteria for the review [13,14,15,16,17]. No relevant studies were identified from the reference lists of the included studies. Figure 1 presents a flowchart of the literature search process.

### 3.2. Characteristics of Studies, Genes/SNPs, and Participants

The characteristics of the 21 studies are summarized in Table 1. All the studies were published between 2008 and 2024, with data collection occurring from November to December 2024. The articles featured populations from diverse countries, with participants originating from Europe (ten studies), Asia (seven studies), North America (two studies), and Brazil (two studies). The number of MM patients in the studies ranged from 40 to 1675. The primary genotyping method employed across the studies was real-time reverse transcriptase–polymerase chain reaction. Table 1 provides details on the total number of participants, number of MM patients, gender distribution, ethnicity, mean age, mean age at diagnosis, genotyping methods, histological confirmation of MM, and key findings from each study. Additionally, Table 2 highlights fou SNPs associated with four genes, with all four SNPs (*DNAH11* rs4487645, *ULK4* rs1052501, *DTNB* rs6746082, and VDR rs1544410) being investigated in more than one study.

### 3.3. Quality Assessment

The methodological quality of the five studies based on the NOS is shown in Table 3. The total score ranged from four to nine stars, with all studies scoring 7. All studies received a star in items 1, 2, 3, and 4 of the “selection” domain, as well as in items 1 and 2 of the “exposure” domain. The “comparability” domain aims to assess whether confounding factors between the case and control groups were identified and adjusted in the analysis so that a maximum of two stars can be alloted in this category. Therefore, none of the analyzed studies received two stars for item 1b; however, all scored for item 1a. The quality of reporting of the included studies based on STREGA is shown in Table 4. The total score ranged from four to eight points, only Martino et al., 2012 [16] obtained the highest score, the other four studies scored 7.

### 3.4. Meta-Analysis

The studies by Broderick et al. (2011) [13], Hongbing Rui et al. (2019) [14], Kumar et al. (2020) [15], Martino et al. (2012) [16], and Weinhold et al. (2014) [17] were selected for meta-analysis.

**Figure 2:**
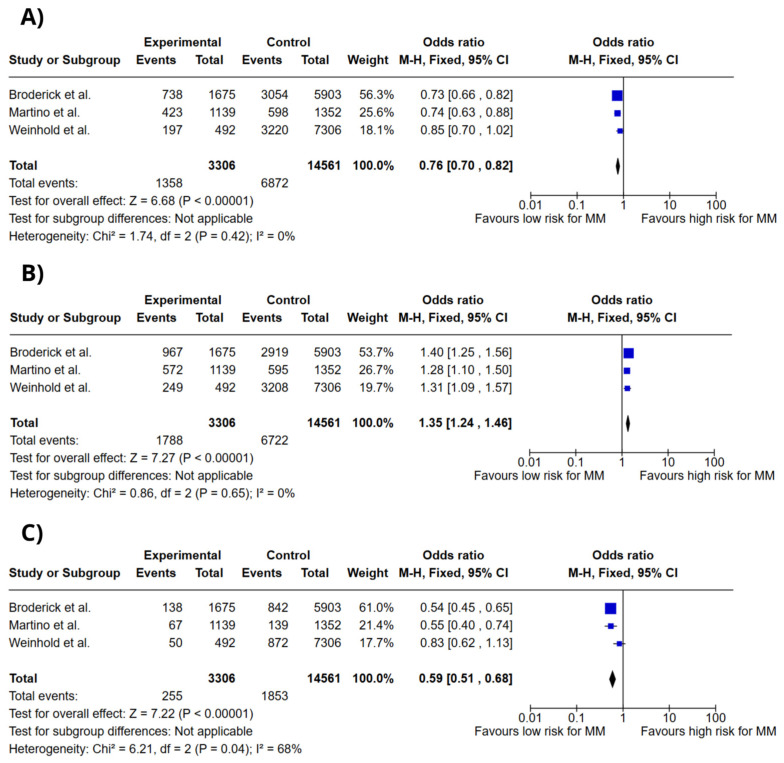
Forest plots of association between *DNAH11* gene polymorphisms and MM risk. (**A**) *DNAH11* rs4487645 C/C genotype. (**B**) *DNAH11* rs4487645 A/C genotype. (**C**) *DNAH11* rs4487645 A/A genotype. Colors do not convey additional information and do not influence data interpretation. Broderick et al. [13], Martino et al. [16], Weinhold et al. [17].

Figure 2 illustrates forest plots for the association between DNAH11 rs4487645 genotypes and multiple myeloma (MM) risk, analyzed under a codominant model. The genotypes assessed included C/C, A/C, and A/A. The meta-analysis revealed a significant association between the DNAH11 rs4487645 A/C genotype and increased risk of MM (OR = 1.35; 95% CI: 1.24–1.46; *p* < 0.00001; I^2^ = 0%). Conversely, the C/C genotype (OR = 0.76; 95% CI: 0.70–0.82; *p* < 0.00001; I^2^ = 0%) and the A/A genotype (OR = 0.59; 95% CI: 0.51–0.68; *p* < 0.00001; I^2^ = 68%) were associated with reduced risk of MM.

**Figure 3:**
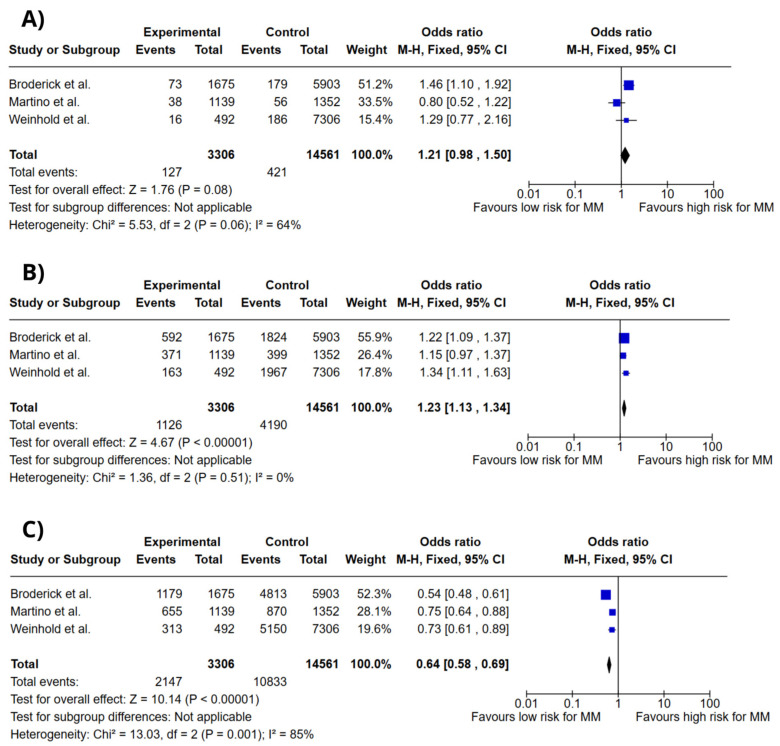
Forest plots of association between *ULK4* gene polymorphisms and MM risk. (**A**) *ULK4* rs1052501 G/G genotype. (**B**) *ULK4* rs1052501 A/G genotype. (**C**) *ULK4* rs1052501 A/A genotype. Colors do not convey additional information and do not influence data interpretation. Broderick et al. [13], Martino et al. [16], Weinhold et al. [17].

Figure 3 depicts forest plots for the association between ULK4 rs1052501 genotypes and MM risk, also analyzed in a codominant model. The genotypes evaluated were G/G, A/G, and A/A. The meta-analysis indicated that the G/G genotype (OR = 1.21; 95% CI: 0.98–1.50; *p* = 0.08; I^2^ = 64%) and the A/G genotype (OR = 1.23; 95% CI: 1.13–1.34; *p* < 0.00001; I^2^ = 0%) were associated with increased risk of MM. However, the A/A genotype was linked to a lower risk (OR = 0.64; 95% CI: 0.58–0.69; *p* < 0.00001; I^2^ = 85%).

**Figure 4:**
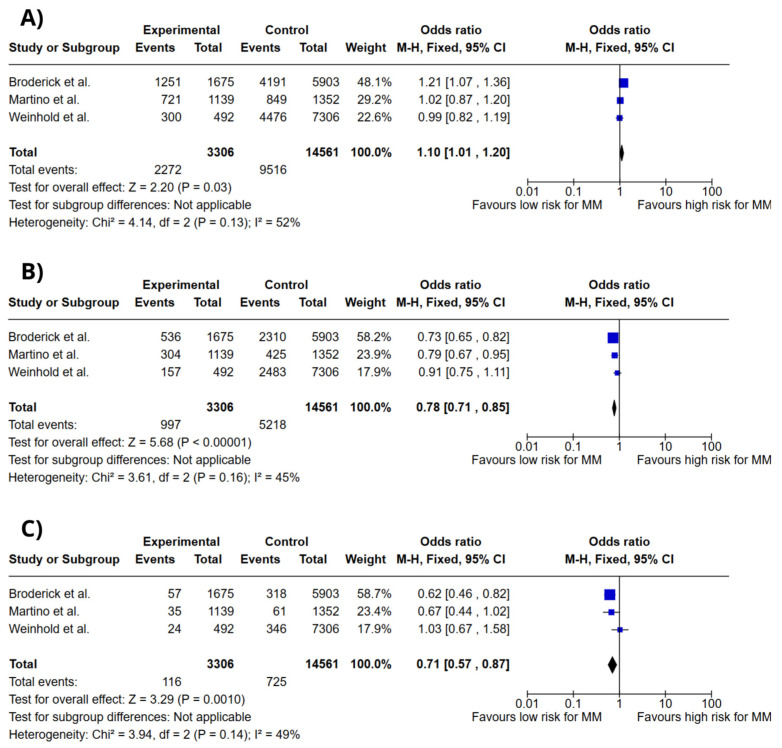
Forest plots of association between *DTNB* gene polymorphisms and MM risk. (**A**) *DTNB* rs6746082 A/A genotype. (**B**) *DTNB* rs6746082 A/C genotype. (**C**) *DTNB* rs6746082 C/C genotype. Colors do not convey additional information and do not influence data interpretation. Broderick et al. [13], Martino et al. [16], Weinhold et al. [17].

Figure 4 presents forest plots for the association between DTNB rs6746082 genotypes and MM risk under the codominant model. The genotypes analyzed were A/A, A/C, and C/C. The meta-analysis showed a modest association between the A/A genotype and increased MM risk (OR = 1.10; 95% CI: 1.01–1.20; *p* = 0.03; I^2^ = 45%). In contrast, the A/C genotype (OR = 0.78; 95% CI: 0.71–0.85; *p* < 0.00001; I^2^ = 45%) and the C/C genotype (OR = 0.71; 95% CI: 0.57–0.87; *p* = 0.001; I^2^ = 49%) were associated with reduced risk of MM.

**Figure 5:**
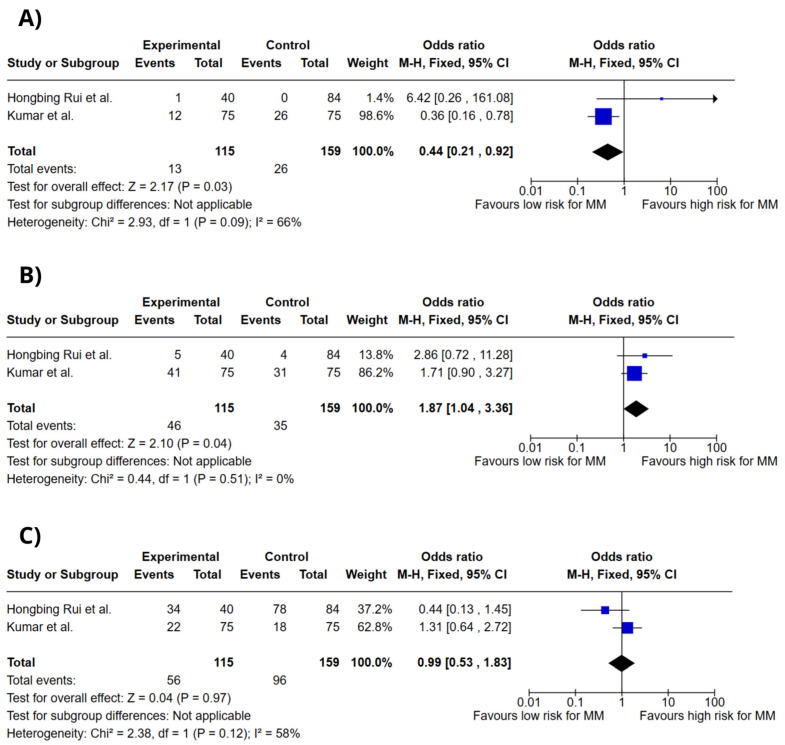
Forest plots of association between *VDR* gene polymorphisms and MM risk. (**A**) *VDR* rs1544410 A/A genotype. (**B**) *VDR* rs1544410 A/G genotype. (**C**) *VDR* rs1544410 G/G genotype. Colors do not convey additional information and do not influence data interpretation. Hongbing Rui et al. [14], Kumar et al. [15].

Figure 5 illustrates forest plots for the association between VDR rs1544410 genotypes and MM risk under the codominant model. The genotypes assessed included A/A, A/G, and G/G. The A/G genotype was significantly associated with increased MM risk (OR = 1.87; 95% CI: 1.04–3.36; *p* = 0.04; I^2^ = 0%). On the other hand, the A/A genotype was associated with reduced risk (OR = 0.44; 95% CI: 0.21–0.92; *p* = 0.03; I^2^ = 66%), while the G/G genotype showed no significant association (OR = 0.99; 95% CI: 0.53–1.83; *p* = 0.97; I^2^ = 58%).

## 4. Discussion

This study represents the first systematic review and meta-analysis to correlate data from the literature with clinically significant genetic variants and genes associated with increased risk of MM. Building upon prior research, we identified genetic variants and candidate genes potentially linked to MM development. Moreover, we established correlations between these allelic variants and the global epidemiological patterns of the disease.

Our analyses revealed significant associations between five genetic variants and susceptibility to MM. Specifically, the *DNAH11* gene variant rs4487645 (A/C genotype), the VDR gene variant rs1544410 (A/G genotype), and the *DTNB* gene variant rs6746082 (A/A genotype) were associated with a heightened risk of MM. Additionally, the *ULK4* gene variant rs1052501 demonstrated a significant association, with its G/G and A/G genotypes linked to an increased risk of MM.

*DNAH11* (dynein axonemal heavy chain 11) encodes a motor ATPase of the dynein heavy chain, a microtubule-dependent protein critical for cilia function [17,34]. This gene has been implicated in primary ciliary dyskinesia [35] and is associated with various malignancies, including esophageal carcinoma [36] and breast cancer [37], underscoring its role in cancer predisposition. The rs4487645 variant is located in intron 80 of the *DNAH11* gene [13] within an 88 kb region of linkage disequilibrium (LD) that also encompasses the 3′ portion of the *CDCA7L* gene. *CDCA7L* interacts with the oncogene *MYC*, functioning as a binding partner of p75 and enhancing MYC’s transformational activity [38]. Given that MYC dysregulation is a hallmark of plasma cell neoplasms, CDCA7L emerges as a strong candidate for the functional basis of the rs4487645 association. Our meta-analysis confirmed that the *DNAH11* rs4487645 A/C genotype is associated with increased MM risk.

*ULK4* (Unc-51-like kinase 4) is located on chromosome 3p22.1 and plays a crucial role in regulating cellular processes such as neurogenesis and intracellular signaling [39]. While it lacks direct kinase activity, ULK4 belongs to the ULK family, which is involved in autophagy-related pathways [39]. It has been identified as a genetic modifier of holoprosencephaly, disrupting Shh signaling and Gli-luc reporter gene expression when knocked down [40]. Recent studies have linked the ULK4 rs1052501 variant with MM, suggesting a role in cell cycle regulation [13,41,42]. Our findings corroborate this association, identifying the rs1052501 G/G and A/G genotypes as risk factors for MM.

*DTNB* (dystrobrevin beta), located on chromosome 2, encodes a protein within the dystrophin-associated protein complex. This complex is critical for maintaining cellular structure and intracellular signaling, particularly in muscle tissues and the brain. *DTNB* is predominantly expressed in neurons of the cortex and hippocampus, where it contributes to early neuronal differentiation [43,44]. Dysfunctional *DTNB* expression has been linked to neurodegenerative conditions such as Alzheimer’s disease and schizophrenia [45]. Additionally, its association with immune cell infiltration highlights its potential role in cancer treatment and prognosis [46]. In our analysis, the *DTNB* rs6746082 A/A genotype was associated with increased MM risk, while the A/C and C/C genotypes appeared protective.

*VDR* (vitamin D receptor) plays a pivotal role in mediating the effects of vitamin D. *VDR* is a nuclear transcription factor that forms heterodimers with RXR (retinoid X receptor) isoforms to regulate gene expression [47]. Polymorphisms in *VDR*, such as rs1544410, may influence vitamin D metabolism and carcinogenesis. Low vitamin D levels, often observed in MM patients, have been linked to cancer progression and associated symptoms such as fatigue and musculoskeletal pain [48]. Our study identified a strong correlation between the *VDR* rs1544410 A/G genotype and increased MM risk, highlighting its relevance in disease susceptibility.

*DNAH11* rs4487645 A/C genotype demonstrated a significant association with increased MM risk, potentially linked to its role in modifying MYC oncogene activity through its interaction with CDCA7L, a well-established MYC-binding partner. Similarly, *ULK4* rs1052501 G/G and A/G genotypes were implicated in MM risk due to their involvement in cell cycle regulation and autophagy-related pathways. *DTNB* rs6746082 A/A genotype’s association with MM risk may stem from its role in maintaining cellular structure and modulating immune responses. Additionally, *VDR* rs1544410 A/G genotype underscores the impact of vitamin D metabolism on MM susceptibility.

Although the identified SNPs demonstrate biological plausibility across populations, their specific impact may differ due to variations in allele frequencies, linkage disequilibrium patterns, and interactions with environmental factors. Additional validation in underrepresented populations is essential to ensure the generalizability and applicability of these findings across diverse ethnicities. Collectively, these polymorphic variants hold promise as predictive biomarkers, offering valuable insights for risk stratification and paving the way for personalized therapeutic strategies in MM management.

## 5. Conclusions

The present study suggests that *DNAH11* rs4487645 A/C genotype, *ULK4* rs1052501 G/G genotype, *ULK4* rs1052501 A/G genotype, *DTNB* rs6746082 A/A genotype, and *VDR* rs1544410 A/G genotype may serve as predictive biomarkers for MM risk. While this study provides valuable insights, some aspects warrant consideration. The focus on previously reported SNPs could overlook unexplored genetic variants, population heterogeneity might influence the generalizability of the findings, and the variants cannot be used to predict the clinical outcome of MM. Future research could address these aspects by conducting larger longitudinal studies, exploring additional genetic variants and SNP-environment interactions, and investigating the molecular mechanisms underlying the role of these SNPs in MM pathogenesis.

## Figures and Tables

**Table 1:** Characteristics of included studies and participants.

Authors, Year	Country	Participants (Male/Female)	Mean Age	Mean Age at Diagnosis (Years)	Genotyping Method	MM Histologically Confirmed	Ethnicity	Main Results
Broderick et al., 2011 [13]	United Kingdom Germany TOTAL	1371 (819/552) 384 (229/155) 1675 (1048/627)	NR NR NR	64.1 ± 10.3 54.5 ± 8.0 NR	Microarray	Yes	European	SNP rs1052501within gene ULK4 and SNP rs4487645 were associated with higher risk of MM
Martino et al., 2012 [16]	Germany	1139	NR	NR	PCR	Yes	European	SNPs rs4487645 and rs6746082 were linked to higher risk of MM
Erickson et al., 2014 [18]	United States of America	1202	57.7	61.0	PCR	NR	European/North American	The SNPs rs12614346 and rs73486634 were linked to an increased risk of MM.
Hosgood III et al., 2008 [19]	United States of America	128 (0/128)	NR	NR	qPCR	Yes	North American	CASP3 and CASP9 polymorphisms were associated with decreased risk of MM.
Karabon et al., 2012 [20]	Poland	200 (94/106)	67 ± 10.9	63.5 ± 11.2	PCR-RFLP	Yes	Danish	Three CTLA variations were identified more frequently in MM patients compared to the control group. Five CTLA polymorphisms were associated with higher risk of MM.
Juan Du et al., 2011 [21]	China	252 (161/91)	58	NR	RT-PCR	NR	Chinese	TRAF3 rs12147254 variant is linked to a reduced risk of developing multiple myeloma, while the rs11160707 genotype has been correlated with improved progression-free survival.
Campa et al., 2011 [22]	German	1188 (643/545)	58.62 ± 10.15	NR	castPCR	Yes	European	Rs2456449 was correlated with the risk of multiple myeloma.
Chubb et al., 2013 [23]	German/United Kingdom	UK-replication-1—812 (412/400) UK-replication-2—396 (181/215) German-replication—1149 (676/473)	NR NR NR	NR 66.0 57.6	PCR KASPar	Yes	European	The variants rs10936599, rs2285803, rs4273077, and rs877529 were correlated with the risk of multiple myeloma.
Martino et al., 2014 [24]	Italy/Poland/Spain/France/Portugal/Hungary/Denmark	1498 (756/742)	60.9 ± 10.6	NR	castPCR	Yes	European	A possible association between the SNP rs2227667 (SERPINE1) and the risk of multiple myeloma in women has been identified.
Faber et al., 2011 [25]	Brazil	150 (81/69)	54	NR	mPCR/ RT-PCR	NR	White people/African-Brazilians	An increased risk of MM was observed in individuals with the VEGF CC genotype combined with GSTM1 undeleted and GSTT1 null genotypes.
Vangsted et al., 2012 [26]	Denmark	348	NR	NR	ABI 7500 or HT7900 systems	NR	Danish	Association between IL1B expression and risk of MM.
Scionti et al., 2022 [27]	Italy	64	NR	NR	DMET Console software version 1.1	NR	Italian	Polymorphism in ADME genes were associated with susceptibility for MM.
Wang et al., 2024 [28]	China	94 (49/45)	59.25 ± 9.36	NR	UE Blood genomic DNA preparation kit	NR	Chinese	Polymorphisms in BNIP3L were associated with susceptibility and prognosis of MM in chinese population.
Hongbing Rui et al., 2019 [14]	China	40 (23/17)	59.5	NR	PCR	NR	Chinese	Association between polymorphisms in VDR gene with increased risk of MM.
Niebudek et al., 2018 [29]	Poland	91 (41/50)	63	NR	PCR-RFLP	NR	Polish	Polymorphism T-129C in ABCB1 gene was not associated with the increased risk of MM development in the polish population.
Kumar et al., 2020 [15]	India	75 (54/21)	57	NR	locus-specific PCR	NR	Indian	FokI, ApaI, and BsmI genotypes were associated with risk of MM in the Indian population.
Peng et al., 2017 [30]	China	827 (473/354)	59.35 ± 9.95	over 60 years	locus-specific PCR	yes	Chinese	rs79480871 were associated with susceptibility for MM.
Li et al., 2019 [31]	China	739 (415/324)	59.27 ± 10.11	older than 65 years	locus-specific PCR	Yes	Chinese	Association for susceptibility in MM by rs61070260 in LRP1B.
Szemraj-Rogucka, 2013 [32]	Poland	174 (93/81)	61	68	PCR-RFLP	NR	Caucasian	Predisposition of MM were associated for rs6449182 of CD38.
Brito et al., 2014 [33]	Brazil	192 (99/93)	62	over 60 years	RT-PCR	NR	Caucasian and Non-Caucasian	Aggressiveness and risk of MM. were associated with VEGF and VEGFR2.
Weinhold et al., 2014 [17]	United Kingdom/Germany	492 (234/258)	67.5	NR	Kompetitive Allele Specific Polymerase (KASP) chain reaction	Yes	European	rs1052501, rs2285803, rs4487645, and rs4273077 increased MM risk.

**Table 2:** Investigated single-nucleotide polymorphisms.

SNPs	Gene	Location	Alleles	Ancestral	Functional Consequence	Clinical Significance
rs1544410	*VDR*	Chromosome 12:47846052	C/A/G/T	C	Intron variant	Benign, likely risk allele
rs6746082	*DTNB*	Chromosome 2:25436375	A/C/G/T	A	Intron variant	NR
rs1052501	*ULK4*	Chromosome 3:41883906	C/G/T	T	Missense variant	Benign
rs4487645	*DNAH11*	Chromosome 7:21898622	C/A/T	C	Intron variant	NR

**Table 3:** Methodological quality of the studies based on the Newcastle–Ottawa Scale (NOS).

Studies	Selection	Item 2	Item 3	Item 4	Comparability	Item 1b	Exposure	Item 2	Item 3	Total Score
	Item 1				Item 1a		Item 1			
Broderick et al., 2011 [13]	*	*	*	*	*		*	*		7
Hongbing Rui et al., 2019 [14]	*	*	*	*	*		*	*		7
Kumar et al., 2020 [15]	*	*	*	*	*		*	*		7
Martino et al., 2012 [16]	*	*	*	*	*		*	*		7
Weinhold et al., 2014 [17]	*	*	*	*	*		*	*		7

* Selection—Item 1: Is the case definition adequate?; Item 2: Representativeness of the cases; Item 3: Selection of controls; Item 4: Definition of controls. Comparability—Item 1a and 1b: Comparability of cases and controls based on the design or analysis. Exposure—Item 1: Ascertainment of exposure; Item 2: Same method of ascertainment for cases and controls; Item 3: Non-response rate.

**Table 4:** The quality of reporting using the Strengthening the Reporting of Genetic Association (STREGA) guideline.

Studies	Description of Genotyping Methods and Errors					Description of Modeling Population Stratification	Description of Modeling Haplotype Variation	Hardy–Weinberg Equilibrium Was Considered	Statement of Whether the Study Is the First Report of a Genetic Association, a Replication Effort, or Both	Score
	Genotyping Methods and Platforms	Error Rates and Call Rates	Genotyping in Batches	Laboratory/Center Where Genotyping Was Performed	The Number of Individuals Successful in Genotyping					
Broderick et al., 2011 [13]	Yes	Yes	Yes	Yes	Yes	Yes	No	Yes	No	7
Hongbing Rui et al., 2019 [14]	Yes	Yes	No	Yes	Yes	Yes	Yes	Yes	No	7
Kumar et al., 2020 [15]	Yes	Yes	Yes	Yes	Yes	Yes	No	Yes	No	7
Martino et al., 2012 [16]	Yes	Yes	Yes	Yes	Yes	Yes	No	Yes	Yes	8
Weinhold et al., 2014 [17]	Yes	Yes	Yes	Yes	Yes	Yes	No	Yes	No	7

## Data Availability

The original contributions presented in this study are included in the article/Supplementary Materials. Further inquiries can be directed to the corresponding author.

## Supplementary Materials

The following supporting information can be downloaded at: https://www.mdpi.com/article/10.3390/ijms26073369/s1.

## Abbreviations

The following abbreviations are used in this manuscript:

MM	Multiple myeloma
SNPs	Single-nucleotide polymorphisms
NR	Not reported
