# Supplementary material for: Single-Nucleotide Polymorphisms Related to Multiple Myeloma Risk: A Systematic Review and Meta-Analysis
Source: Int J Mol Sci. 2025 Apr 4;26(7):3369. doi: 10.3390/ijms26073369 (PMC11989572; doi:10.3390/ijms26073369)
Supplement: Supplementary file 1 [file ijms-26-03369-s001.zip › ijms-3414216-supplementary.pdf]

Table S1. Search strategies

Database	Search strategy
PubMed (n= 4)	(((((((((("Multiple Myeloma"[MeSH Terms]) OR ("Multiple Myelomas")) OR ("Myelomas, Multiple")) OR ("Myeloma, Plasma-Cell")) OR ("Myeloma, Plasma Cell")) OR ("Myelomas, Plasma-Cell")) OR ("Plasma-Cell Myeloma")) OR ("Plasma-Cell Myelomas")) OR ("Myeloma-Multiple")) OR ("Myeloma Multiple")) OR ("Myeloma-Multiples")) OR ("Myeloma, Multiple")) OR ("Plasma Cell Myeloma")) OR ("Cell Myeloma, Plasma")) OR ("Cell Myelomas, Plasma")) OR ("Myelomas, Plasma Cell")) OR ("Plasma Cell Myelomas")) OR ("Kahler Disease")) OR ("Disease, Kahler")) OR ("Myelomatosis")) OR ("Myelomatoses") AND (((((((("Genetic Predisposition to Disease"[MeSH Terms]) OR ("Genetic Susceptibility")) OR ("Genetic Susceptibilities")) OR ("Susceptibilities, Genetic")) OR ("Susceptibility, Genetic")) OR ("Genetic Predisposition")) OR ("Genetic Predispositions")) OR ("Predispositions, Genetic")) OR ("Predisposition, Genetic") OR (((((("Polymorphism, Single Nucleotide"[MeSH Terms]) OR ("Nucleotide Polymorphism, Single")) OR ("Nucleotide Polymorphisms, Single")) OR ("Polymorphisms, Single Nucleotide")) OR ("Single Nucleotide Polymorphisms")) OR ("SNPs")) OR ("Single Nucleotide Polymorphism") AND (("Risk")[MeSH Terms]) OR (("Risk"[Title/Abstract]))) OR (("Risks"[Title/Abstract]))) OR (("Relative Risk"[Title/Abstract]))) OR (("Relative Risks"[Title/Abstract]))) OR (("Risk, Relative"[Title/Abstract]))) OR (("Risks, Relative"[Title/Abstract])))
Web of Science (n= 27)	((((((((((TS=("Multiple Myeloma")) OR TS=("Multiple Myelomas")) OR TS=("Myelomas, Multiple")) OR TS=("Myeloma, Plasma-Cell")) OR TS=("Myeloma, Plasma Cell")) OR TS=("Myelomas, Plasma-Cell")) OR TS=("Plasma-Cell Myeloma")) OR TS=("Plasma-Cell Myelomas")) OR TS=("Myeloma-Multiple")) OR TS=("Myeloma Multiple")) OR TS=("Myeloma-Multiples")) OR TS=("Myeloma, Multiple")) OR TS=("Plasma Cell Myeloma")) OR TS=("Cell Myeloma, Plasma")) OR TS=("Cell Myelomas, Plasma")) OR TS=("Myelomas, Plasma Cell")) OR TS=("Plasma Cell Myelomas")) OR TS=("Kahler Disease")) OR TS=("Disease, Kahler")) OR TS=("Myelomatosis")) OR TS=("Myelomatoses") AND (((((((((((((((ALL=("Genetic Predisposition to Disease")) OR ALL=("Genetic Susceptibility")) AND ALL=("Genetic Susceptibilities")) OR ALL=("Susceptibilities, Genetic")) OR ALL=("Susceptibility, Genetic")) OR ALL=("Genetic Predisposition")) OR ALL=("Genetic Predispositions")) OR ALL=("Predispositions, Genetic")) OR ALL=("Predisposition, Genetic")) OR ALL=("Polymorphism, Single Nucleotide")) OR ALL=("Nucleotide Polymorphism, Single")) OR ALL=("Nucleotide Polymorphisms, Single")) OR ALL=("Polymorphisms, Single Nucleotide")) OR ALL=("Single Nucleotide Polymorphisms")) OR ALL=("SNPs")) OR ALL=("Single Nucleotide Polymorphism") AND ((ALL=("Risk")) OR ALL=("Risks")) OR ALL=("Relative Risk")) OR ALL=("Relative Risks")) OR ALL=("Risk, Relative")) OR ALL=("Risks, Relative"))
Scopus (n= 365)	TITLE-ABS-KEY ("Multiple Myeloma") OR TITLE-ABS-KEY ("Multiple Myelomas") OR TITLE-ABS-KEY ("Myelomas, Multiple") OR TITLE-ABS-KEY ("Myeloma, Plasma-Cell") OR TITLE-ABS-KEY ("Myeloma, Plasma Cell") OR TITLE-ABS-KEY ("Myelomas, Plasma-Cell") OR TITLE-ABS-KEY ("Plasma-Cell Myeloma") OR TITLE-ABS-KEY ("Plasma-Cell Myelomas") OR TITLE-ABS-KEY ("Myeloma-Multiple") OR TITLE-ABS-KEY ("Myeloma Multiple") OR TITLE-ABS-KEY ("Myeloma-Multiples") OR TITLE-ABS-KEY ("Myeloma, Multiple") OR TITLE-ABS-KEY ("Plasma Cell Myeloma") OR TITLE-ABS-KEY ("Cell Myeloma, Plasma") OR TITLE-ABS-KEY ("Cell Myelomas, Plasma") OR TITLE-ABS-KEY ("Myelomas, Plasma Cell") OR TITLE-ABS-KEY ("Plasma Cell Myelomas") OR TITLE-ABS-KEY ("Kahler Disease") OR TITLE-ABS-KEY ("Disease, Kahler") OR TITLE-ABS-KEY ("Myelomatosis") OR TITLE-ABS-KEY ("Myelomatoses")AND TITLE-ABS-KEY ("Genetic Predisposition to Disease") OR TITLE-ABS-KEY ("Genetic Susceptibility") AND TITLE-ABS-KEY ("Genetic Susceptibilities") OR TITLE-ABS-KEY ("Susceptibilities, Genetic") OR TITLE-ABS-KEY ("Susceptibility, Genetic") OR TITLE-ABS-KEY ("Genetic Predisposition") OR TITLE-ABS-KEY ("Genetic Predispositions") OR TITLE-ABS-KEY ("Predispositions, Genetic") OR TITLE-ABS-KEY ("Predisposition, Genetic") OR TITLE-ABS-KEY ("Polymorphism, Single Nucleotide") OR TITLE-ABS-KEY ("Nucleotide Polymorphism, Single") OR TITLE-ABS-KEY ("Nucleotide Polymorphisms, Single") OR TITLE-ABS-KEY ("Polymorphisms, Single Nucleotide") OR TITLE-ABS-KEY ("Single Nucleotide Polymorphisms") OR TITLE-ABS-KEY ("SNPs") OR TITLE-ABS-KEY ("Single Nucleotide Polymorphism") AND TITLE-ABS-KEY ("Risk") OR TITLE-ABS-KEY ("Risks") OR TITLE-ABS-KEY ("Relative Risk") OR TITLE-ABS-KEY ("Relative Risks") OR TITLE-ABS-KEY ("Risk, Relative") OR TITLE-ABS-KEY ("Risks, Relative")
